# Ultrastructural features of neuroblastic tumours in relation to morphological, and molecular findings; a retrospective review study

**DOI:** 10.1186/1472-6890-14-13

**Published:** 2014-03-31

**Authors:** Elizabeth Latimer, Glenn Anderson, Neil James Sebire

**Affiliations:** 1 Institute of Child Health, UCL, London, UK; 2 Department of Histopathology, UCL, London, UK; 3 Great Ormond Street Hospital for Children Foundation Trust, London WC1N 3JH, UK

**Keywords:** Neuroblastoma, Ultrastructure, Electron microscopy, Molecular biology, MYCN

## Abstract

**Background:**

Neuroblastoma is the most common solid tumour of infancy and is responsible for 15% of childhood cancer deaths. Presence of amplified *MYCN* in neuroblastoma is associated with poor prognosis and rapid tumour progression. The aim of this study was to examine and compare the ultrastructural features of high-risk *MYCN* amplified neuroblastomas, with lower-risk non-*MYCN* amplified tumours.

**Methods:**

This was a retrospective study evaluating archival diagnostic tissue samples, in which Fluorescence in-situ hybridisation (FISH) had been used at diagnosis to establish *MYCN* status. 22 (11 *MYCN* amplified tumours and 11 non-*MYCN* amplified) tumours of similar light microscopic appearance (poorly differentiated neuroblastoma) were then selected for ultrastructural examination.

**Results:**

There is a relationship between ultrastructural features in neuroblastoma and *MYCN* status, although with marked overlap between groups. *MYCN* amplified tumours generally exhibited a ‘less differentiated’ ultrastructural phenotype, with significantly smaller neurotubules (NT) in the cell body (p < 0.002). Non-*MYCN* amplified tumours show increased features of neuronal differentiation, with fewer neurosecretory granules (NSG) and NT in the cytoplasm.

**Conclusions:**

*MYCN* amplification is associated with a less differentiated ultrastructural phenotype, and lack of *MYCN* amplification with relative ultrastructural neuronal differentiation.

## Background

Neuroblastoma is the most common solid tumour of childhood, and with an incidence of 1 in 10,000, it is responsible for 15% of childhood cancer deaths [1,2]. Neuroblastoma originates from primordial neural crest cells, often occurring in sympathetic ganglia and chromaffin cells of the adrenal medulla; however it can occur anywhere along the sympathetic chain, including the pelvis, neck and brain [3]. Neuroblastoma is classed as one of the ‘*small blue round cell tumours*’ and by routine light microscopy demonstrates sheets and nests of small primitive appearing cells with speckled, hyperchromatic nuclei, scant cytoplasm and poorly defined cell borders. The nested background demonstrates faintly eosinophillic fibrillary neuropil, a mass of interwoven nerve endings, dendrites and other neurone components [4].

Prognosis can vary markedly, from fatal to spontaneous regression [1,5]. Molecular biology findings are associated with adverse prognosis, the best studied of which is amplification of the *MYCN* oncogene. *MYCN* is a member of the *MYC* proto-oncogene family, and is a nuclear transcription factor not normally found in adult tissues. [6,7]. Presence of *MYCN* amplification is associated with adverse outcome and tumour progression [8,9].

Ultrastructural features of neuroblastic tumours are well-described [10-14] but there are no data regarding ultrastructural findings specifically in relation to *MYCN* status. This study examines the ultrastructure of high-risk *MYCN* amplified neuroblastomas, compared with low-risk non-*MYCN* amplified tumours to test the hypothesis that neuroblastomas will have ultrastructural differences related to their molecular characteristics.

## Methods

A search of the histopathology diagnostic archive was made to identify neuroblastomas (Neuroblastic tumour, neuroblastoma (Schwannian stroma poor), poorly differentiated subtype’ based on light microscopic assessment) with potentially viable material stored for electron microscopic examination and *MYCN* status assessment performed as part of routine clinical care. 25 *MYCN* amplified, and 25 matched non-*MYCN* amplified, neuroblastomas were initially identified, of which 22, 11 cases in each group, were suitable for ultrastructural analysis. Suitability was judged based on the amount of necrosis present and tumour preservation observed at ultrastructural level. The number of cases was considered statistically significant for the study with a power of 80% to detect differences of 25% between groups.

Samples were initially confirmed as poorly differentiated neuroblastomas by light microscopy using 3 μm formalin fixed paraffin embedded tissue and a Haematoxylin and Eosin stain. Sectioning and staining were carried out by standard methods as per standard [14]. Further confirmation of neuroblastoma was by the use of immunohistochemistry for NB84 and CD56 [4]. Fluorescence *in-situ* Hybridisation (FISH) was performed as part of routine clinical care using a standard protocol [15]. We used a commercial probe available from Abbott Molecular, USA, with each new batch validated in the laboratory before use on patient material. (Vysis LSI N-MYC (2p24) SpectrumGreen/CEP 2 SpectrumOrange Probe (Abbott Molecular, USA)). Cells hybridized using this dual-colour probe show multiple green signals when MYCN amplification is present. When investigating MYCN status by Fluorescence In Situ Hybridization (FISH), MYCN amplification is defined as >4-fold MYCN signals compared to 2p reference probe signals. MYCN amplification can occur as either intrachromosomal homogeneously staining regions (HSRs) or as genetically unstable extrachromosomal double minutes (DMs) [16].

For ultrastructural analysis, samples were fixed in 2.5% glutaraldehyde in 0.1 M cacodylate buffer with secondary fixation in 1.0% osmium tetroxide. Tissues were dehydrated in graded ethanol and transferred to propylene oxide and finally infiltrated and embedded in Agar 100 epoxy resin. 90 nanometre ultra-thin sections were cut using a Diatome diamond knife on a Leica Ultracut UCT ultramicrotome. Sections were picked up on copper grids and stained with alcoholic uranyl acetate and Reynold’s lead citrate. The samples were then viewed with JEOL (UK) Ltd, JEM-1400 120 kV Transmission Electron Microscope. All measurements were carried out using the JEOL microscope and Advanced Media Technologies Inc (AMT) software and were recorded with the images. Initial observations included overall tumour architecture, cell adhesion and stroma and subsequent quantitative observations included the most characteristic and frequently seen features of neuroblasts, cytoplasmic (neuritic) processes and the neurosecretory granules (NSG). The cell processes usually contain neurotubules (NT), and intermediate filaments [17]. The position of these tubules within the cell, and whether the tubules were in the cytoplasm, or within the processes was observed. The number and diameters (nm) of the neurotubules (NT) present in the cell body were measured in multiple well preserved and viable cells per sample (total 45 measurements in the 11 MYCN amplified group and 48 measurements in the 11 non-MYCN amplified group). NSG are small, round, membrane bound secretory granules, which contain a characteristic central dense core surrounded by a peripheral halo [18]. The number of NSG present in the cell body were counted and their diameter measured. The numbers of the NSG and NT were measured only within the cell body, as it was not possible to track the cell processes using a 2-D plane. All diameter measurements were carried out using the AMT software and all measurements were in nanometres (nm).

All statistical analyses were calculated with SPSS (Statistical Package for the Social Sciences), version 19.0, IBM, UK. The parametric student *T*-test and the non-parametric Mann-Whitney *U* test were used to compare distributions between groups as appropriate. All results were considered statistically significant at p < 0.05. Ethical approval was obtained from the National Research Ethics Service (NRES) committee London, Bloomsbury (REC reference number 11/LO/1175). Approval was gained from Great Ormond Street NHS Hospital Foundation Trust and Institute of Child Health (UCL) (R&D number 10MH31) and the study was funded by Great Ormond Street Children’s Charity (V0907). All cases used were non-identifiable, archival cases.

## Results

**Figure 1:**
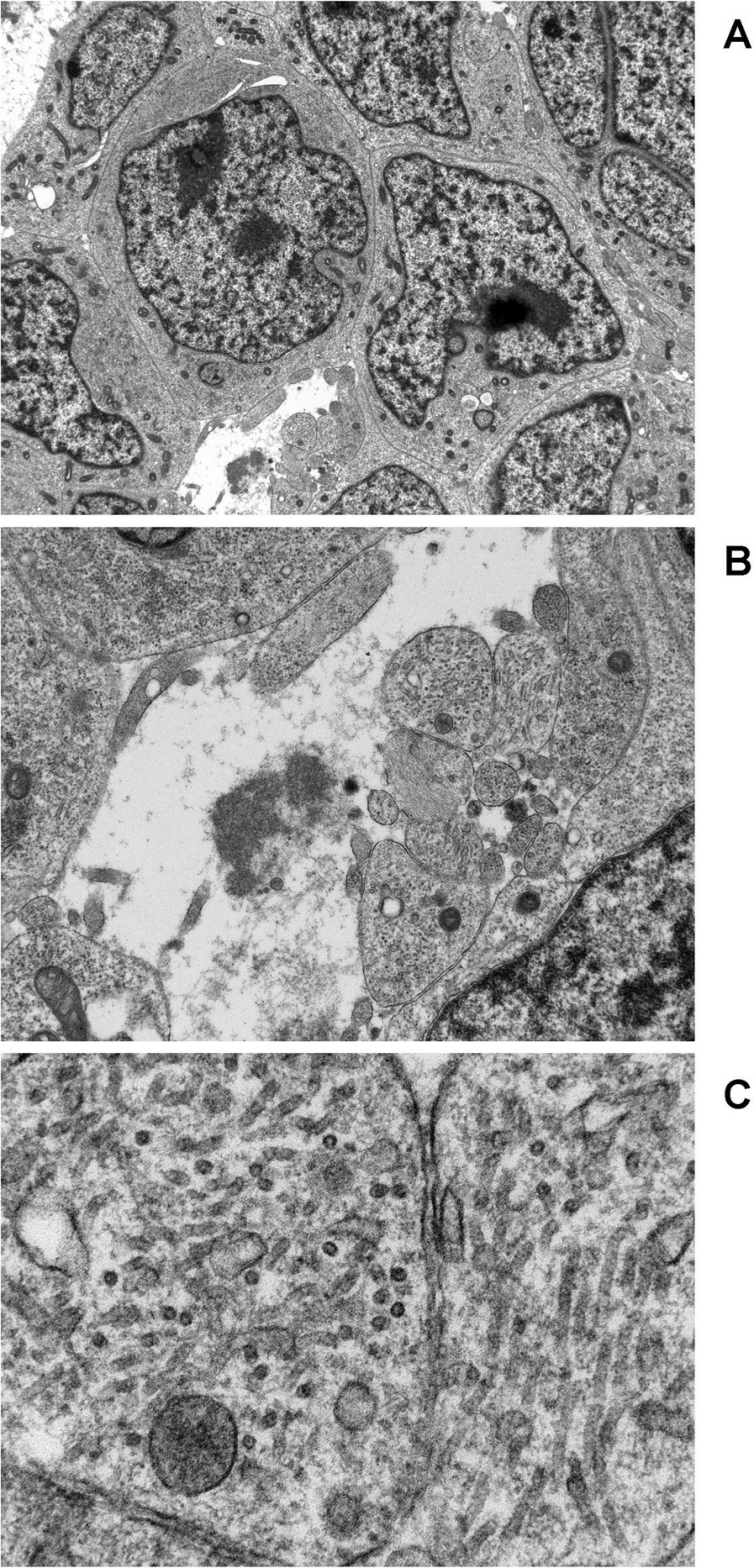
**Photomicrographs of MYCN amplified neuroblastoma demonstrating: A**. Poorly differentiated neuroblasts with irregular nuclear outline and moderately sized nucleoli. The cytoplasm is scant and contains a few mitochondria and numerous polyribosomes with no evidence of cell junctions. Neuritic (cytoplasmic) processes are sparse and occasional neurosecretory granules are present in the cell cytoplasm. Original magnification ×1500. **B**. Rudimentary neurites are present in this area of the micrograph with numerous microtubules but no neurosecretory granules, emphasising the lack of differentiation. Often solitary mitochondria are also seen in the cytoplasmic process. Original magnification ×5000. **C**. Higher magnification shows microtubules in transverse and longitudinal orientation. Their mean diameter was 22.5 nm. Original magnification ×25000.

**Figure 2:**
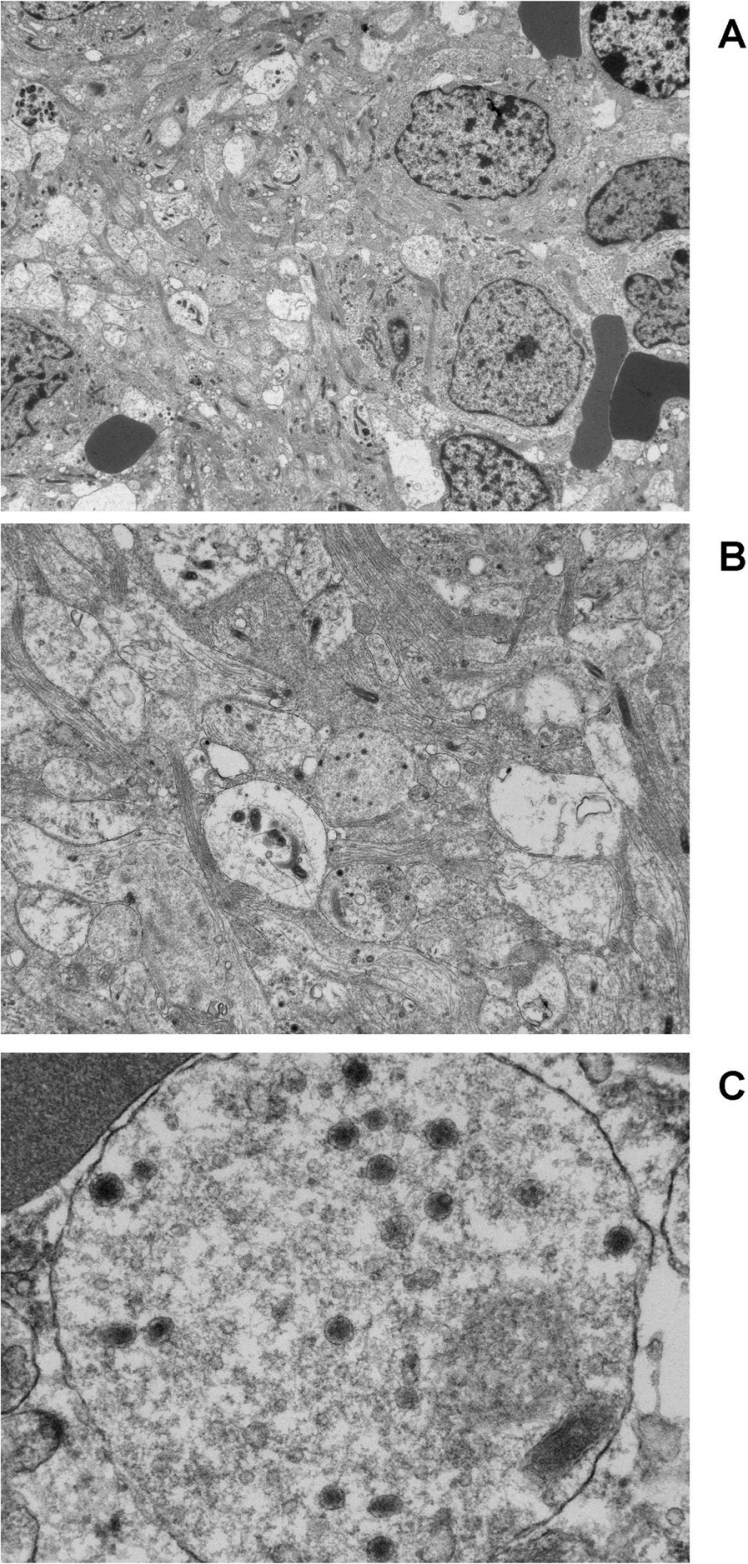
**Photomicrographs of non-MYCN amplified neuroblastoma demonstrating: A**. Neuroblastoma with widespread neuritic processes. Tumour cells are small and round and do not show ganglion cell differentiation at this stage. Original magnification ×1000. **B**. The image demonstrates abundant sheaves of microtubules and neurites in transverse section with many electron dense neurosecretory granules. Original magnification ×3000. **C**. A high power view of a neurite process with dense-core, neurosecretory granules with a characteristic peripheral halo. The mean diameter of the granules in all cases was 98 nm. Original magnification ×15000.

The mean median age of patients across both groups was 27 (range 1-144) months, with no significant difference in age between the *MYCN* amplified and non-amplified groups (P = 0.55). Ultrastructural morphological examination demonstrated that *MYCN* amplified tumours had a subjectively ‘less differentiated’ ultrastructural appearance, with scanty neuritic processes, which contained fewer NSG and NT. The non-*MYCN* amplified tumours had a more differentiated ultrastructural appearance, with frequent cell processes all appearing well developed with abundant NSG and NT, (Figures 1 and 2).

**Figure 3:**
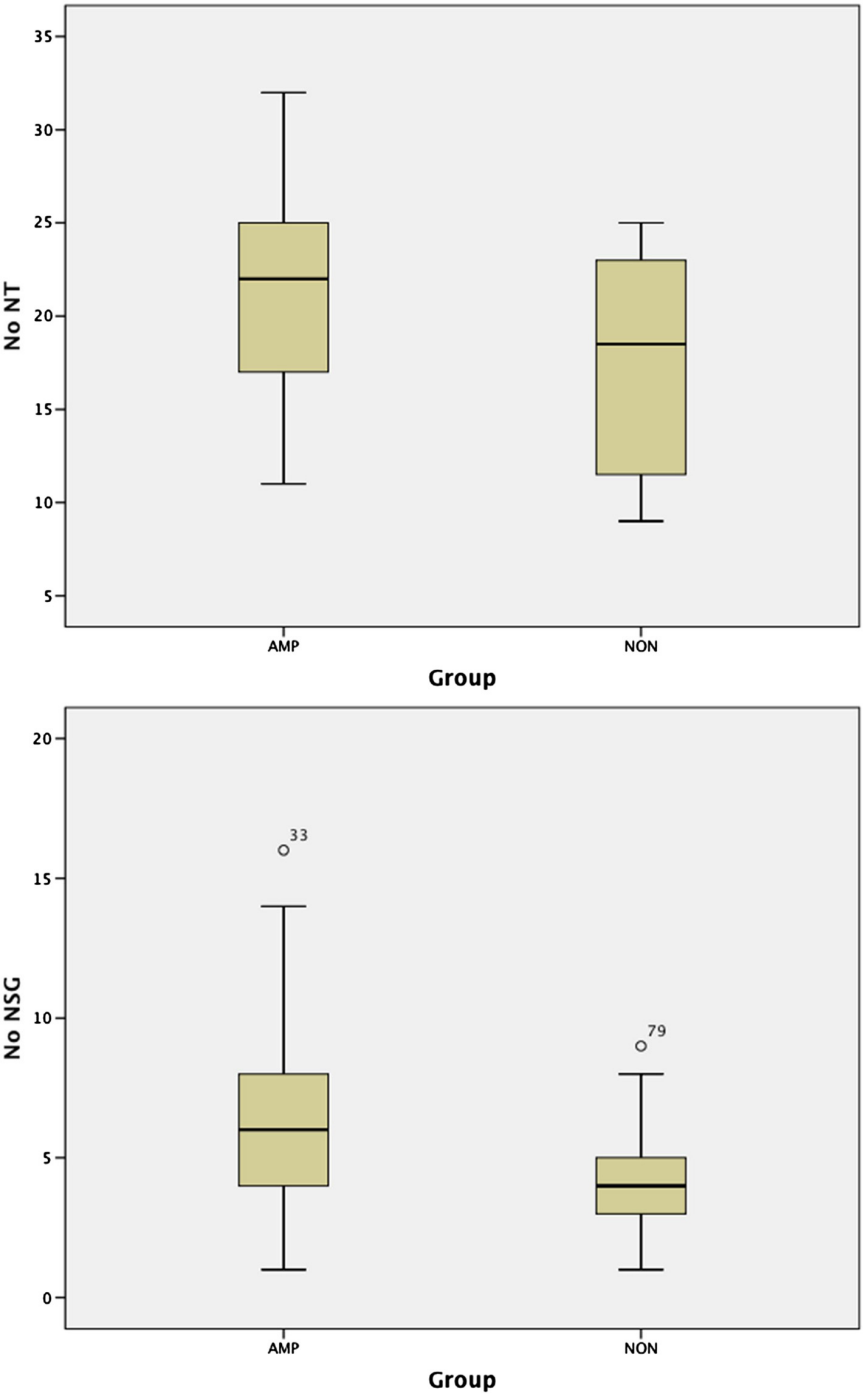
Box-whisker plots demonstrating distribution of number of neurotublues (NO NT) and number of neurosecretory granules (NO NSG) per cell examined in MYCN amplified (AMP) versus non-MYCN amplified (NON) morphologically poorly differentiated neuroblastomas.

There were significantly fewer NSG and NT in the cell body of the non-*MYCN* amplified neuroblastomas, but with markedly overlapping distributions. The mean number of NSG in the cell body was 6.4/cell for the *MYCN* amplified group versus 4.1/cell for the non-*MYCN* amplified group (P < 0.001). The mean number of NT in the cell body of the *MYCN* amplified group was 21.8/ cell compared to a mean of 17.7/cell for the non- *MYCN* amplified group (P < 0.001). The mean NSG diameters were not significantly different between groups. (P = 0.88), mean diameter of all cases, 98 nm. However, the mean diameter of NT in the *MYCN* amplified group was significantly less than the non-*MYCN* amplified group (22.5 nm versus 23.8 nm respectively (P < 0.002) (Figure 3).

## Discussion

The findings of this study have demonstrated that there are significant ultrastructural morphological differences between *MYCN*-amplified and non-*MYCN*-amplified neuroblastomas, including a statistically significant reduction in diameter of NT, and significantly more NSG and NT in the cell body of *MYCN-*amplified tumours. These differences may be explained by the finding that *MYCN* amplified cells appear less differentiated, and therefore the NSG and NT in the cell bodies have not yet extended into cell processes. Additionally, since *MYCN*-amplified tumours appear less differentiated, the smaller size of their NT may represent tubules in these tumours being less well-developed.

*MYCN* is involved in the expression of many target genes, which regulate a range of cellular processes, including cell growth, proliferation, differentiation, and apoptosis [19]. *MYCN* is primarily an embryonic developmental gene, whose normal expression is specifically restricted (in humans and mice) to certain tissues in the developing embryo. *MYCN* is specifically found in the neuroepithelium, and also in the developing lung, heart kidney and intestine [7,20]. Primary functions of *MYCN* are proposed to be related to control of cell proliferation and enabling cells to remain in relatively undifferentiated states [19]. Over expression of *MYCN* in the neural crest results in an increased generation of neurons but down regulation of *MYCN* is required for these neurons to become terminally differentiated [21,22]. In combination, these findings suggest that *MYCN* signalling is important in maintaining cells in an undifferentiated state and that down regulation of *MYCN* can lead to neuronal differentiation [6]. The present ultrastructural findings support these suggested roles since *MYCN* amplified cells had a morphologically less differentiated appearance, with associated morphometric changes.

Increased *MYCN* expression keeps cells in an undifferentiated and proliferative state [6]. Therefore, the hypothesis has been raised that blocking *MYCN* expression or its action could lead to less aggressive tumours, and lead to new therapies for high-risk patients [23]. Differentiation as a target for therapy has been investigated and several groups are attempting to down regulate *MYCN* expression as a treatment in high-risk *MYCN* amplified neuroblastoma. Several agents have been investigated, of which 13-cis-retinoic acid shows the most promise, showing neurite outgrowth and differentiation of human neuroblastoma cells *in vitro* and *in vivo*[6]. These results are in accordance with the current ultrastructural findings, that lower-risk tumours show substantial neurite outgrowth and were morphologically more differentiated, despite being within the poorly differentiated light microscopy subgroup of neuroblastomas.

The present study demonstrated a significant difference in both the size and number of neurotubules within *MYCN* amplified tumours compared to the non-*MYCN* amplified tumours, which has not been reported previously. Microtubules and intermediate filaments, of which neurotubules are an example, are involved in intracellular transport and it can be hypothesised that if a cell has a more patent network of tubules and filaments then drug transport may occur more efficiently. This remains entirely speculative but may offer a possible explanation regarding the association of *MYCN* amplified tumours with adverse outcome.

Whilst markers of differentiation, such as dense core neurosecretory granules and neuritic processes, may be identified, and the neuropil seen on light microscopy is identified as neuritic processes with NSG, poorly differentiated neuroblastomas have previously been reported to generally show no major ultrastructural variations related to outcome not identified by light microscopy, although there may be an association between low numbers of NSG and adverse clinical course [10-13].

## Conclusions

There are ultrastructural morphological differences in neuroblastoma, dependent upon *MYCN* amplification status. The data is limited by the small study size but provides the first data in this area, and is consistent with current evidence on functioning of *MYCN*, with increased *MYCN* expression being associated with a less differentiated phenotype.

## Competing interests

The authors declare that they have no competing interests.

## Authors’ contributions

BL and GA carried out the electron microscopic examination and interpretation. NJS, BL and GA participated in the design of the study and performed the statistical analysis. NJS conceived of the study, and participated in its design and coordination and helped to draft the manuscript. All authors drafted, read and approved the final manuscript.

## Pre-publication history

The pre-publication history for this paper can be accessed here:

http://www.biomedcentral.com/1472-6890/14/13/prepub
